# Intra-hospital transport of adult critically ill patients treated with high flow nasal cannula oxygen: a prospective observational multicenter study

**DOI:** 10.1186/s13613-025-01502-7

**Published:** 2025-07-08

**Authors:** Mai-Anh Nay, Alice Bisson, Adrien Auvet, Agathe Delbove, Aziz Berrouba, Toufik Kamel, Maxime Desgrouas, Thierry Boulain

**Affiliations:** 1 Médecine Intensive Réanimation, Centre Hospitalier Universitaire d’Orléans, Orléans, France; 2 Département d’anesthésie-réanimation, Centre Hospitalier Régional Universitaire de Tours, Tours, France; 3 Réanimation Médico-Chirurgicale, Centre Hospitalier de Dax, Dax, France; 4 Service de réanimation polyvalente, Centre Hospitalier Bretagne Atlantique, Vannes, France; 5 Réanimation polyvalente, Centre Hospitalier de Martigues, Martigues, France

**Keywords:** High flow nasal cannula, Oxygenation, Intra-hospital transport, Intensive care unit, Critical ill patient

## Abstract

**Background:**

Acute respiratory failure is a common reason for admission to the intensive care unit, and patients are frequently treated with high-flow nasal cannula oxygen therapy (HFNC). Intra-hospital transport of critically ill patients, such as between hospital wards and the intensive care unit or for diagnostic exams, is common. Transportable HFNC can be used during these intra-hospital transports. We aimed to evaluate the complications associated with intra-hospital transport of patients treated with HFNC.

**Methods:**

We conducted a prospective, descriptive multicenter study between May 2022 and May 2024, involving critically ill adult patients who were treated with HFNC prior to transport and required intra-hospital transport for any reason, accompanied by an intensive care unit team. The primary objective was to evaluate the incidence of severe adverse events including severe hypoxemia (with pulse oxygen saturation of less than 80%), need for intubation, need for non-invasive ventilation or cardiorespiratory arrest during transport. Secondary objectives were to assess the incidence of non-severe adverse events, defined as the need for increased inspired oxygen fraction, switching HFNC for standard oxygen therapy, nasal cannula removal or dysfunction of the HFNC device.

**Results:**

We included 165 patients and analyzed 187 transports. Eight (4.3%) severe adverse events occurred in 7 patients including 6 cases of severe transient hypoxemia and 2 cases of non-invasive ventilation. All of them were transient severe hypoxemia that occurred during the first transport. Forty-three (23%) non-severe adverse events occurred, including 29(15.6%) cases of increased inspired oxygen fraction requirement, 7/187 (3.74%) cases of nasal cannula removal, 6/187 (3.2%) cases of HFNC device dysfunction, and 1 (0.5%) case involved replacing HFNC with standard oxygen therapy.

**Conclusion:**

HFNC during intra-hospital transport of critically ill patients had a low incidence of severe adverse events. Non-severe adverse events were more frequent, but their potential impact could not be assessed in this study and warrants further investigation.

*Trial registration*: Clinicalstrials.gov, NCT05311007, registered 23 March 2022, https://clinicaltrials.gov/study/NCT05311007?term=hospiflow&rank=1.

**Supplementary Information:**

The online version contains supplementary material available at 10.1186/s13613-025-01502-7.

## Background

Acute respiratory failure is one of the primary reasons for hospitalization in intensive care units (ICUs) [1, 2].

High-flow nasal cannula oxygen therapy (HFNC) is commonly used in the ICU, particularly for patients with hypoxemic acute respiratory failure [3]. It can also be used in cases of chronic obstructive pulmonary disease exacerbation or post-extubation [3]. HFNC delivers humidified and heated gas with a precise fraction of inspired oxygen (FiO_2_) ranging from 21 to 100%, providing better patient comfort and a slight positive expiratory pressure [4].

Intra-hospital transport of critically ill patients is frequent and exposes them to severe adverse events, occurring in 4–33% of transports, depending on the population studied. These events are often related to the severity of the patients’ condition [5, 6]. The most recent French multicenter study reported a 15% complication rate in critically ill patients across different ventilation modalities [7].

Intra-hospital transport may be required for diagnostic or therapeutic reasons, such as transfer to the radiology department, the operating room, or between hospital wards and the ICU. During transport, ventilatory support must be maintained. If a patient is already receiving HFNC in a hospital ward and requires transfer to an ICU not equipped with a transportable HFNC device, he or she must be switched to a high-concentration oxygen mask or intubated.

Transportable HFNC could reduce transport-related risks and provide a safe alternative for transferring patients with acute respiratory failure to or from the ICU. Studies, mostly in pediatric inter-hospital transport, have shown a decreased need for mechanical ventilation and minimal adverse effects with transportable HFNC use. However, no such evaluation exists for critically ill adult patients. This study aimed to assess the safety of intra-hospital transport of critically ill adult patients treated with HFNC during transport.

## Methods

### Study design and population

We conducted a prospective descriptive study in four ICUs in France between May 2022 and May 2024. Adult patients aged ≥ 18 years who were treated with HFNC prior to transport and required intrahospital transport for any reason (e.g., imaging, operating room, transfers from emergency department (ED) or ward to ICU, transfers from ICU to ward) accompanied by an ICU team (one nurse and one physician or resident) were systematically identified and enrolled. Exclusion criteria included inter-hospital transports, patients without French social security, those under legal protection, and pregnant or breastfeeding women.

The study protocol was approved by the French Ethics Committee (Comité de Protection des Personnes—Ile de France VI—No. 22.00476.000051) on March 8, 2022, and registered at Clinicaltrials.gov (NCT05311007). The study was conducted in accordance with the Declaration of Helsinki. Patients, or, if they were unable to provide consent, their next of kin, gave oral informed consent.

### Modalities for transport

All patients were on HNFC prior to each study inclusion, whether in the ED, ward, or ICU. For transport, a dedicated transportable HNFC was used. The nasal cannulas remained in place with immediate disconnection from the initial HNFC device and reconnection to the transportable HNFC. Following inclusion in the study, patients received standard care during transport, accompanied by an ICU team. This included continuous monitoring of heart rate, pulse oxygen saturation, respiratory rate, and blood pressure. Blood pressure was monitored continuously if the patient had an arterial catheter or every 5 min using an automated brachial cuff, if applicable. HFNC settings were determined at the clinician’s discretion.

Regarding power supplies, in one center, energy packs with a 6-h battery life and a weight of 12 kg were used (Valise energie 230 V 1460 W h, Laberine Energie, Orchies, France) with the AIRVO 2 device (Fisher & Paykel Healthcare, New Zealand). In the other centers, portable AIRVO 2 units with specific batteries providing 30–40 min of autonomy with a weight of 18 kg were used (Trippe Lite Smartpro 700 VA 450 W, Cleveland, United States). In both cases, the power supplies and HFNC were fixed on a wheeled stand (eFigure 1A, B).

### Outcomes

The primary objective was to estimate the incidence of severe adverse events during intrahospital transport of critically ill patients. Severe adverse events were defined as the need for intubation during transport, the need for non-invasive ventilation, severe hypoxemia (with pulse oxygen saturation [SpO_2_] < 80%), or the occurrence of cardiorespiratory arrest during transport. Secondary objectives included assessing the incidence of non-severe adverse events during these transports. These were defined as the need to increase FiO_2_, nasal cannula removal, switching HFNC for standard oxygen therapy or dysfunction of the HFNC device (e.g. battery failure or oxygen delivery failure) during transport. All transports that occurred from inclusion to day 28 in the ICU were recorded.

### Data collection

Standardized paper forms were used to record the following data: patient baseline characteristics, reason for intra-hospital transport, transport duration, and the most recent blood gas measurements. Respiratory rate, arterial blood pressure, heart rate, SpO_2_, and HFNC settings (flow and FiO_2_) were recorded 5 min before (baseline), during transport (starting with the use of the transportable HFNC), and 5 min after transport. The ROX index, defined as the ratio of SpO_2_/FiO_2_ to respiratory rate, was also calculated based on the previously recorded parameters, at the same timepoints [8]. Each case report form was then transcribed into an electronic file (Excel, Microsoft Corporation, Redmond, WA).

### Sample size considerations

Given the lack of prospective data in adults on the intrahospital transfer of patients receiving HFNC, we chose a convenience sample of 100–200 patients across four centers over a 24-month period. We had planned to stop the study either upon reaching 200 patients or at the predefined study end date.

### Statistical analysis

Categorical variables are presented as numbers and percentages. Continuous variables are reported as medians with 25th and 75th percentiles (interquartile range [IQR]).

We planned to investigate predictors of severe adverse events, as well as the combined occurrence of severe and non-severe adverse events during the first transport, using logistic regression. Patient clinical characteristics present in more than 5% of the study population, or accounting for more than 5% of all adverse events, and associated with the dependent variable at *P* < 0.2 in bivariate analysis were included in a multivariable logistic regression model in a single step. Adjusted odds ratios (OR) are given with their 95% confidence intervals (95%CI).

Linear mixed-effects modeling was used to assess the evolution of SpO_2_, respiratory rate, heart rate, and arterial blood pressure during all recorded transports, considering patients as having random and correlated intercepts and slopes.

Several categories were defined according to (1) SpO_2_/FiO_2_ ratio: SpO_2_/FiO_2_ ≤ 235, SpO_2_/FiO_2_ > 235 and ≤ 315, and SpO_2_/FiO_2_ > 315 [9]; (2) indication for transport: ICU-Radiology round-trip for imaging (including computed tomography scan, positron emission tomography scan), from ED or ward to ICU.

A two-tailed *P* < 0.05 was considered statistically significant. All analyses were performed using R software version 4.2.3 [10].

## Results

**Fig. 1:**
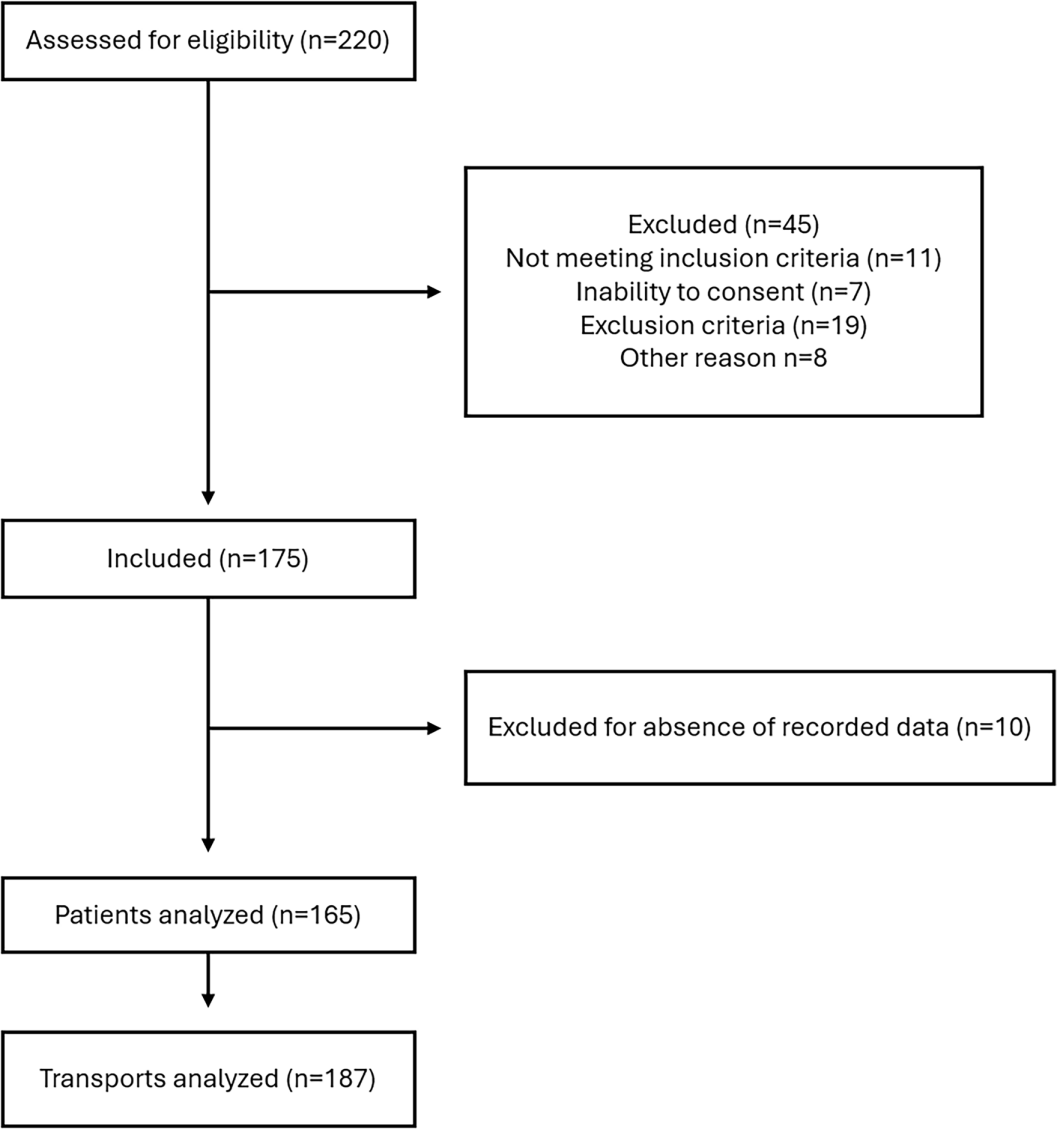
Flow chart

During the study period, 175 patients were included, and 10 were subsequently excluded due to the absence of recorded data. A total of 165 patients were analyzed, accounting for 187 transports, as some patients underwent multiple transports (Fig. 1; eTable 1). There were 56 women and 109 men, with a median age of 69 years [IQR 59–74] and a body mass index of 26 [IQR: 23–30]. Other patient characteristics are shown in Table 1. For the 1st transport, 127 (77%) patients had a SpO_2_/FiO_2_ ≤ 235, 30(18.2%) a SpO_2_/FiO_2_ > 235 and ≤315 and 8(4.8%) a SpO_2_/FiO_2_ > 315 (Table 1). Over all 187 transports, 143 (76.5%) had a SpO_2_/FiO_2_ ≤ 235, 35(18.5%) a SpO_2_/FiO_2_ > 235 and 9(4.8%) a SpO_2_/FiO_2_ > 315 (eTable 1). HFNC settings at baseline were an FiO_2_ of 0.5 [IQR 0.4–0.6] and a median flow rate of 50 L/min [IQR 50–60], with 31 patients receiving a flow rate ≤ 40 L/min.

**Table 1:** Characteristics

Variables	All patients (*N* = 165)
Age (years), median (IQR)	69 (59–74)
Sexe—Female, *n* (%)	56 (33.9)
BMI (kg/m^2^), median (IQR)	26 (23–30)
SAPS II, median (IQR)	41 (32–51)
Reasons for admission^a^	
Surgery within 24 h after admission, *n* (%)	4 (2.4)
Postoperative, *n* (%)	7 (4.2)
Acute respiratory failure, *n* (%)	134 (81.2)
Acute on chronic respiratory failure, *n* (%)	38 (23.0)
Coma, *n* (%)	4 (2.4)
Septic shock, *n* (%)	9 (5.5)
Cardiogenic shock, *n* (%)	3 (1.8)
Myocardal infarction, *n* (%)	4 (2.4)
Acute kidney failure, *n* (%)	13 (7.9)
Acute liver failure, *n* (%)	1 (0.6)
Infections	
Pulmonary, *n* (%)	107 (64.8)
Abdomen, *n* (%)	6 (3.6)
Urinary, *n* (%)	2 (1.2)
Comorbidities	
Active smoking, *n* (%)	38 (23.0)
Weaned smoking, *n* (%)	33 (20.0)
Pulmonary restrictive syndrome, *n* (%)	19 (11.5)
Chronic obstructive pulmonary disease, *n* (%)	37 (22.4)
Emphysema, *n* (%)	13 (7.9)
Asthma, *n* (%)	17 (10.3)
Interstitial lung injury, *n* (%)	12 (7.3)
Sleep apnea disease, *n* (%)	17 (10.3)
Non-invasive home ventilation, *n* (%)	15 (9.1)
Long-term oxygen therapy, *n* (%)	12 (7.3)
Hypertension, *n* (%)	79 (47.9)
Ischemic heart disease, *n* (%)	29 (17.6)
Chronic heart failure, *n* (%)	3 (1.8)
Atrial fibrillation, *n* (%)	21 (12.7)
Chronic kidney disease, *n* (%)	20 (12.1)
Cirrhosis, *n* (%)	6 (3.6)
Immunosuppression, *n* (%)	36 (21.8)
SOFA score prior to the first transport	3 (2–5)
*Baseline respiratory parameters before the first transport*	
SpO_2_/FiO_2_ ratio at baseline, *n* (%)	
SpO_2_/FiO_2_ ≤ 235	127 (77)
SpO_2_/FiO_2_ > 235 and ≤315	30 (18.2)
SpO_2_/FiO_2_ > 315	8 (4.8)
Initial SpO_2_/FiO_2_, median (IQR)	
ICU-radiology round-trip for imaging	196 (157–241)
From ED or ward to ICU	139 (100–167)
Other transfers^b^	223 (182–240)
Initial ROX index, median (IQR)	
ICU-radiology round‑trip for imaging	8.4 (6.2–10.7)
From ED or ward to ICU	5.2 (4.1–7.6)
Other transfers^b^	9.6 (8.5–11.8)

*BMI* body mass index, *SAPS II* simplified acute physiology score, *IQR* interquartile range, *SOFA* Sequential Organ Failure Assessment, *SpO*_*2*_ pulse oxygen saturation of oxygen, *FiO*_*2*_ inspired fraction of oxygen, *ED* emergency department, *ICU* intensive care unit

^a^Reasons could be multiple

^b^Included coronary angiography, radiography, to or from operating room, Doppler ultrasound, ICU to medical ward

The most recent blood gas measurement before the first transport had a median arterial partial pressure of oxygen (PaO_2_) of 74 mmHg [IQR 63–88] and a median arterial partial pressure of carbon dioxide (PaCO_2_) of 36 mmHg [IQR 32–44]. The oxygen flow rate at the time of the most recent arterial blood gas analysis, which could have been performed under either standard oxygen therapy or HFNC, had a median of 50 L/min [IQR: 15–60]. Among patients who were on HFNC at the time of the blood gas, the median FiO_2_ was 0.5 [IQR: 0.4–0.6]. The most recent blood gas measurements before the second transport are shown in the Appendix (eTable 2).

The number of transports per patient is described in Table 2. The duration of the first transport was less than 10 min for 47 patients (28.5%), between 10 and 20 min for 53 patients ( (32.1%) and over 20 min for 65 patients 39.4%) (Table 2). The duration of the second transport was less than 10 min for 3 patients (16.7%), between 10 and 20 min for 7 (38.9%) and over 20 min for 8 patients (44.4%).

**Table 2:** Characteristics of transport

Variables	All patients (*N* = 165)
**Transports per patient**	
Had a single transport, *n* (%)	147 (89.1)
Had two transports, *n* (%)	15 (9.1)
Had three transports, *n* (%)	2 (1.2)
Had four transports, *n* (%)	1 (0.6)
**Indication of first transport**	
ICU-radiology round‑trip for imaging	84 (50.9)
From ED or ward to ICU	56 (33.9)
Other transfers, *n* (%)^a^	25 (15.1)
**Duration of first transport** (*N* = 165)	
<10 min, *n* (%)	47 (28.5)^b^
10–20 min, *n* (%)	53 (32.1)^c^
>20 min, *n* (%)	65 (39.4)^d^
**Duration of all transports** (*N* = 187)	
<10 min, *n* (%)	52 (27.8)^e^
10–20 min, *n* (%)	60 (32.1)^f^
>20 min, *n* (%)	75 (40.1)^g^

*ED* emergency department, *ICU* intensive care unit

^a^Included coronary angiography, radiography, to or from operating room, doppler ultrasound, ICU to medical ward

^b^37 patients went from ED or ward to ICU, 1 for ICU-radiology round-trip for imaging and 9 for others

^c^17 patients went from ED or ward to ICU, 26 for ICU-radiology round-trip and 10 for others

^d^2 patients went from ED or ward to ICU, 57 for ICU-radiology round-trip and 6 for others

^e^37 concerned transfer from ED or ward to ICU, 3 concerned ICU-radiology round trip for imaging and 12 concerned other transfers

^f^17 concerned transfer from ED or ward to ICU, 28 concerned ICU-radiology round trip for imaging and 15 other transfers

^g^2 concerned transfer from ED or ward to ICU, 61 concerned ICU-radiology round trip for imaging and 12 other transfers

The main reasons for first transport were ICU-radiology round trip for imaging (50.9%), transfers from ED or ward to the ICU (33.9%), and other transfers (15.1%) (Table 2). The main reasons for other transports are shown in the Appendix (eTable 2).

### Outcomes

Severe adverse events occurred in 8 out of 187 cases (4.3%), all during the first transport. Six (3.2%) were episodes of severe hypoxemia (SpO_2_ < 80%) lasting less than 5 min and two (1.1%) involved a switch to non-invasive ventilation. Overall, severe adverse events occurred in seven patients (4.2%); one patient experienced both severe hypoxemia and required non-invasive ventilation. No cases of cardiac arrest or intubation occurred during intrahospital transport (Table 3).

**Table 3:** Outcomes in all transports

Variables	All transports (*N* = 187)
**Severe adverse events**, *n* (%)^a^	8 (4.3)
Severe hypoxemia*, *n* (%)	6 (3.2)
Use on non-invasive ventilation^b^, *n* (%)	2 (1.1)
Cardia arrest, *n* (%)	0
Intubation, *n* (%)	0
**Non-severe adverse events**, %	43 (23.0%)
Increased of FiO_2_, *n* (%)^c^	29 (15.6)
Nasal cannula removal, *n* (%)^d^	7 (3.7)
Device dysfunction, *n* (%)^e^	6 (3.2)
HFNC replaced by standard O_2_ therapy, *n* (%)^f^	1 (0.5)

*FiO*_*2*_ inspired fraction of oxygen, *HFNC* high flow nasal cannula

* Defined by a pulse oxygen saturation of oxygen under 80% (SpO_2_ < 80%)

^a^All occurred during the first transport

^b^One patients had severe hypoxemia and use of NIV during the first transport. Therefore, the number of transports during which at least one severe adverse event occurred was 7/187 (3.7%)

^c^26 occurred during the 1st transport, 1 during the 2nd transport and 2 during the 3rd transport

^d^6 occurred during the 1st transport and 1 during the 3rd transport. Four cases were due to human error, involving oxygenation failure from an empty oxygen cylinder, and two were due to device malfunctions (battery failure)

^e^Included discharged battery or oxygen delivery system failure. 5 occurred during the 1st transport and 1 during the 2nd transport

^f^In this case, the patient was transported for MRI imaging, for which HFNC was not compatible. Standard oxygen was initiated prior to entering the MRI suite, and HFNC was resumed immediately after the examination for the return transfer to the ICU. No complications, such as desaturation, occurred during transport or the imaging procedure

A total of 43 (23%) non-severe adverse events were recorded during the 187 intra-hospital transfers examined, including 29 cases (15.6%) of increased FiO_2_ requirements, 7 cases (3.74%) of nasal cannula removal, 6 cases (3.2%) of device dysfunction, and 1 case(0.5%) involved a switch to standard oxygen therapy (Table 3). The team proactively increased FiO_2_ before transport in 5 patients (2.6%) which was not considered an adverse event. However, in one of these cases, FiO_2_ was decreased 5 min into the transport but subsequently had to be increased again due to clinical need; this was therefore classified as an adverse event.

Overall, 36 patients (21.8%) experienced at least one severe or non-severe adverse event during transport (34 patients (20.6%) did so during the first transport) (eTable 3). FiO_2_ was significantly more frequently increased during transport for imaging (24/92) compared to the two other categories (5/95) (*P* < 0.0001) (eTable 4). Across all transports, 27 out 165 patients (including 25 during the first transport) had at least one desaturation episode (SpO_2_ < 90%) (eTable 5).

### Evolution of SpO_2_, respiratory parameters, neurological status, HFNC settings and arterial blood pressure

**Fig. 2:**
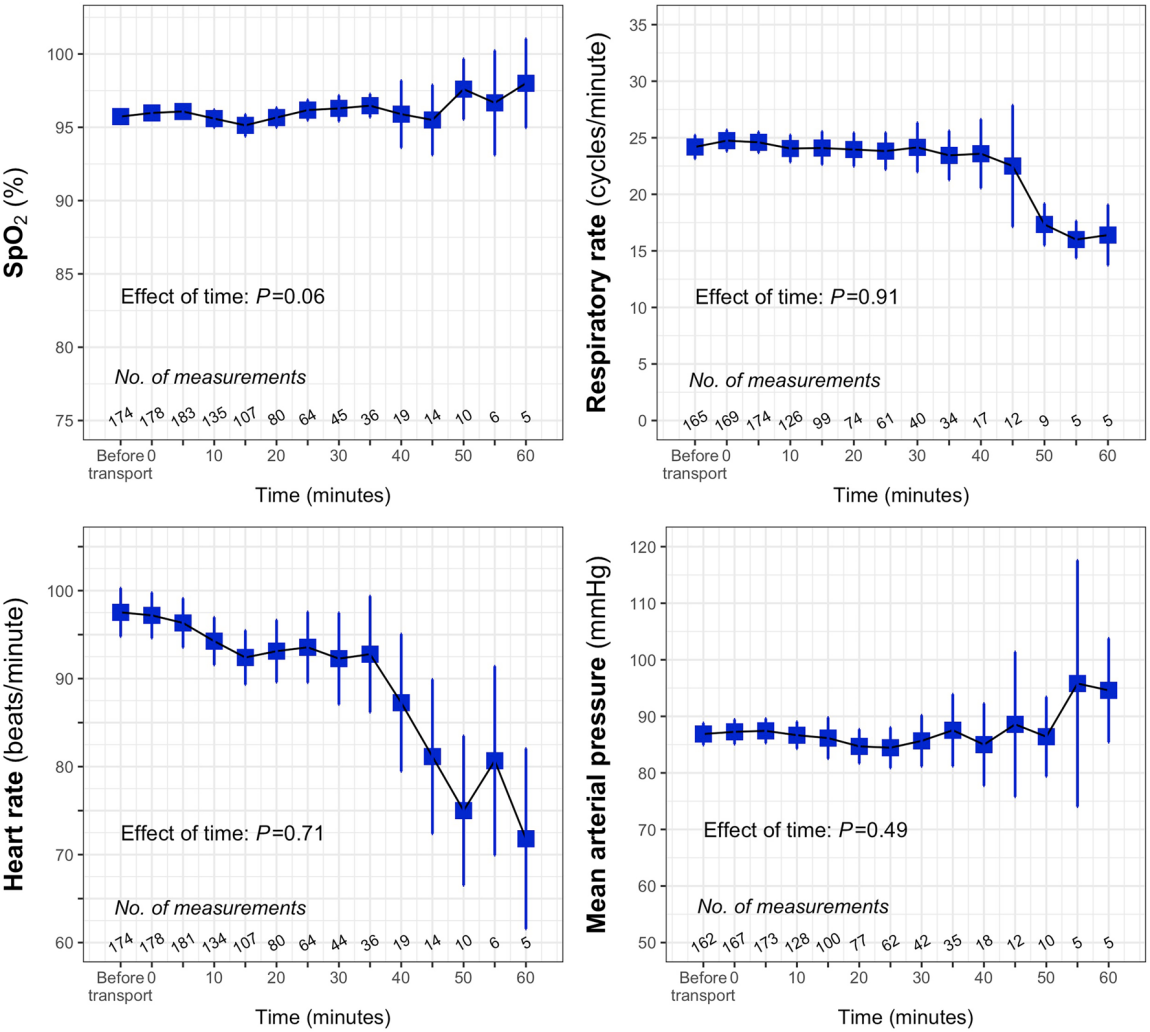
Evolution of SpO_2_, respiratory rate, heart rate and mean arterial blood pressure during transport. Blue square boxes indicate the mean value, with vertical bars representing the 95% confidence interval. Values at the “before transport” time point were recorded within 5 min prior to transport. The analysis was restricted to the first 60 min as very few transports lasted longer than 1 h. The number of measurements indicates the data available at each time point. Note that there were some missing values (e.g., 178 SpO_2_ values at time 0 out of 187 transports) that were not replaced. SpO_2_, pulse oxygen saturation

Overall, SpO_2_, respiratory rate, heart rate and arterial blood pressure remain steady during transport (Fig. 2; eFigure 2). Regarding hemodynamics, 5 patients received norepinephrine, all during the first transport. In every case, norepinephrine was initiated prior to transport, and no dose escalation was required (with initial data missing for one patient). The mean dose before transport was 0.88 mg/h (±1.02) and the mean dose after transport was 0.74 mg/h (±0.68). Across all transports, no deterioration in neurological status, assessed using the Glasgow Coma Scale, was observed in 97.9% of cases (183/187). All neurological deteriorations occurred during the first transport. Additionally, five patients showed improvement in their Glasgow score during the first transport.

**Fig. 3:**
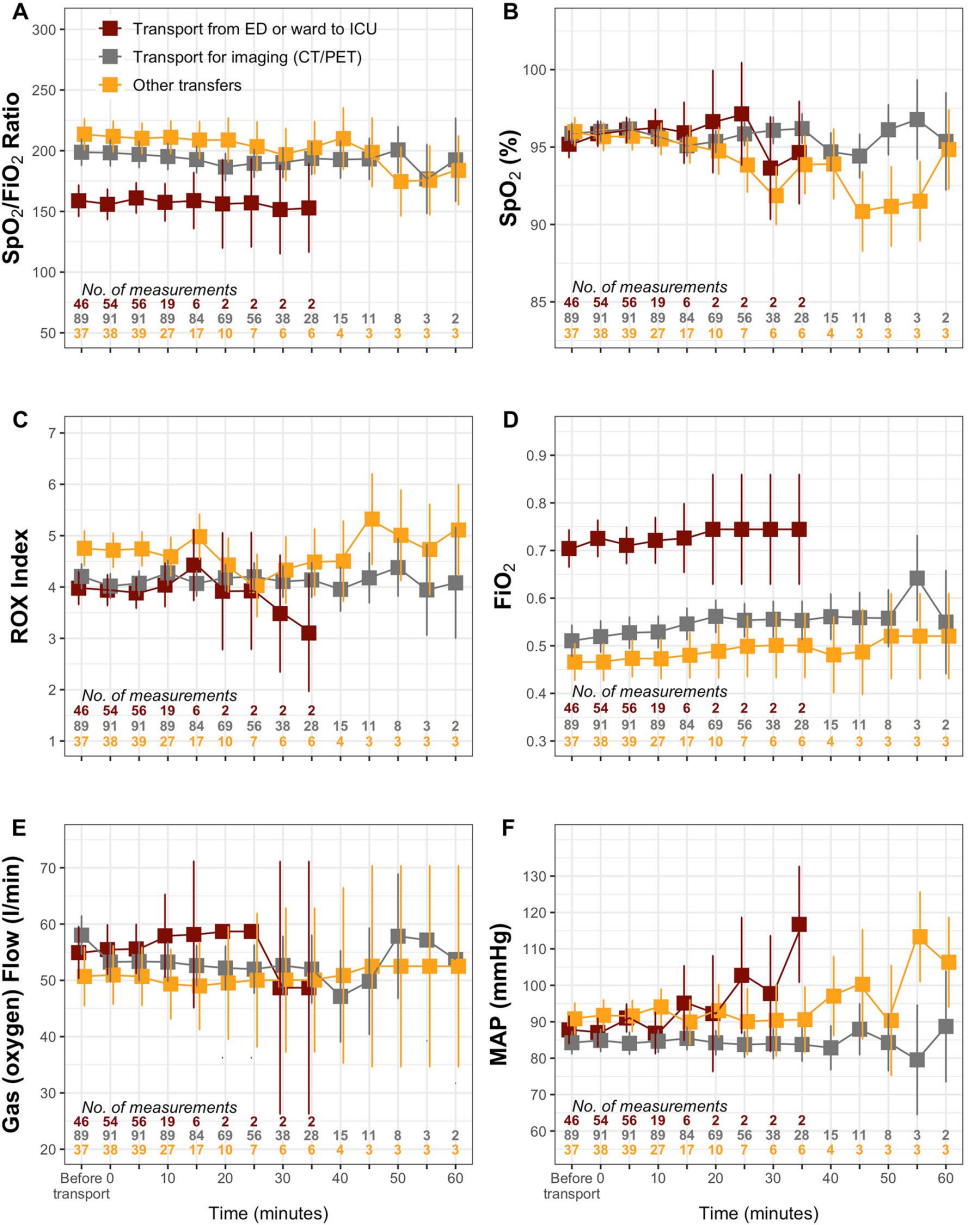
Evolution of SpO_2_/FiO_2_ ratio, SpO_2_, ROX index, HFNC settings (FiO_2_ and flow rate), and mean arterial blood pressure during transport, compared across the three categories of transport. Red square boxes indicate the transport from ED or ward to ICU. Grey square boxes indicate the transport for imaging (CT/PET). Yellow square boxes indicate other transports. Squares indicate estimated means and errors bars represent the 95% confidence interval of the mean. Values at the “before transport” time point were recorded within 5 min prior to transport. **A** Linear mixed‐effects model results: Baseline (before transport) SpO_2_/FiO_2_ ratio was significantly lower in patients transported from the ward or the ED than in patients transported for imaging and lower than in patients transported for other reasons (*P* < 0.05). Baseline SpO_2_/FiO_2_ ratio was also significantly lower in patients transported for imaging than in patients transported for other reasons (*P* < 0.05). There was no significant transport × time interaction (*P* > 0.05), indicating that the between‐group differences observed at baseline remained essentially constant throughout the 60-min observation period. **B** Linear mixed‐effects model results: There were no baseline differences in SpO_2_ between types of transport (*P* > 0.05), but a significant transport × time interaction (*P* < 0.05). Divergence between groups became significant at 15 min; this finding should be interpreted cautiously given the small number of patients remaining at later time points. **C** Linear mixed‐effects model results: There was no significant interaction between time and type of transport. At baseline (before transport) both the ROX index of patients transported for imaging and of those transported from the ward or the ED were significantly lower than the ROX index of patients transported for other reasons (*P* < 0.05 for both). There was no significant transport × time interaction (*P* > 0.05), indicating that the between‐group differences observed at baseline remained essentially constant throughout the 60-min observation period. **D** Linear mixed‐effects model results: FiO_2_ at baseline (before transport) was significantly higher in patients transported from the ward or the ED than in patients transported for imaging and higher than in patients transported for other reasons (*P* < 0.05). This was also significantly higher in patients transported for imaging than in patients transported for other reasons (*P* < 0.05). There was no significant transport × time interaction (*P* > 0.05), indicating that the between‐group differences observed at baseline remained essentially constant throughout the 60-min observation period. **E** Linear mixed‐effects model results: There were no significant differences in gas flow settings across the different types of transport at baseline (before transport). There was no significant transport × time interaction (*P* > 0.05). **F** Linear mixed‐effects model results: Baseline MAP (before transport) was significantly lower in patients transported for imaging than in patients transported for other reasons (than transport from the ward or from the ED) (*P* < 0.05). There was a significant transport x time interaction (*P* < 0.05). MAP in patients transported from the ward or from the ED was higher than MAP in patients transported for imaging from time 0 to time 35 min (*P* < 0.05 at each time point). Throughout the entire 60 min period, MAP in the imaging group remained lower than in the other-transfers group (all *P* < 0.05). SpO_2_, pulse oxygen saturation; FiO_2_, inspired fraction of oxygen; HFNC, high flow nasal cannula; MAP, mean arterial blood pressure; ED, emergency department; ICU, intensive care unit; CT, computed tomography; PET, positron emission tomography

The evolution of SpO_2_/FiO_2_, ROX index, HFNC settings and arterial blood pressure according to transport indications are shown in Fig. 3.

### Prediction of adverse events

Severe adverse events were too few to allow a meaningful search for predictor variables using logistic regression, as originally planned.

The factors associated with the occurrence of an adverse event (either severe or non-severe) during the first transport, as identified in the bivariate analysis, are presented in eTable 6 of the supplementary file. Two multivariable analyses were conducted the first one including variables selected by univariate analysis (c.f. methods); the second one was, in addition, adjusted for variables intended to reflect overall disease severity and the severity of respiratory impairment. In both models (eTables 7, 8), transport for imaging was the sole variable significantly with severe or non-severe adverse event occurrence.

## Discussion

In this prospective, observational, multicenter study, 4.3% of intra-hospital transports of patients treated with HFNC were associated with severe adverse events, and 23% with non-severe adverse events. Severe complications were rare and predominantly due to severe hypoxemia. Non-severe complications, observed in approximately one-quarter of transports, were mostly an increased FiO_2_ requirement. Transport for imaging was associated with an increased risk of any adverse events.

These findings are consistent with previous studies evaluating intra-hospital transport with other oxygenation or ventilation modes, suggesting that HFNC does not imply additional risks compared to other modalities. The incidence of complications during intra-hospital transport of mechanically ventilated patients varies widely in the literature. For example, one study reported a 33% complication rate when considering all types of adverse events [6], while another, focusing on perioperative patients (70% mechanically ventilated), reported a lower rate of 4.2% [5]. In the latter, adverse events such as desaturation were defined more stringently, requiring a ≥5% decrease from baseline lasting over 5 min, and FiO_2_ increases were not classified as complications. A recent French study reported a 15% overall complication rate, with only 6.5% of cases involving spontaneously breathing patients (receiving non-invasive ventilation or high-concentration oxygen mask) [7].

To date, retrospective studies on HFNC use during transport have primarily focused on pediatric inter-hospital transfers. One study found that the implementation of HFNC increased the use of HFNC and was not inferior to standard oxygen or non-invasive ventilation [11]. These studies reported increased FiO_2_ or flow rate in 25–75% of cases and demonstrated the safety of HFNC, with minimal risk of escalation to non-invasive ventilation, no requirement for intubation, and potential reductions in pediatric ICU length of stay and duration of respiratory support [12–16]. In adults, only case reports documented the use of HFNC during inter-hospital transfer [17–19].

The use of HFNC for intra- or inter-hospital transport in adults must consider energy source and oxygen consumption [20]. In our study, centers were equipped with AIRVO2 devices featuring external batteries with autonomy ranging from 30–40 min to up to 6 h in the largest center, which utilized an energy kit.

Among the six cases of device dysfunction observed in our study, four were attributed to oxygenation failure due to an empty oxygen cylinder, and two to device malfunctions (battery failure). Transfers were conducted using oxygen cylinders, with reconnection to the wall source immediately after arrival in the ICU, the operating room or the radiology department. All four device failures resulted from human error, underlying the critical importance of team training and expertise in managing these transfers.

Transport for imaging was associated with an increase of any adverse events. These results may be explained by the fact that transports for imaging are generally longer in duration. Additionally, patients are required to remain in a strict supine position during the examination, which may alter pulmonary ventilation/perfusion ratios[21–23].

HFNC is known to reduce the work of breathing, with higher flow rates generally associated with greater physiological benefit. In our study, the median baseline flow rate was 50 L/min [IQR: 50–60] [24, 25]; however, 8% of patients received a flow rate ≤ 40 L/min, which was likely less effective. It is not possible, based on the available data, to determine whether these lower flow settings were the result of clinical decision-making or reflected patient discomfort with HFNC in the potentially stressful context of transport.

Our study has several limitations. First, it was conducted in centers already equipped with HFNC, where teams were familiar with its use. Although a mix of academic and non-academic centers was included, most patients were enrolled in a single center. This may limit the generalizability of the results to other centers with different levels of experience or protocols. In addition, team expertise during transport likely influences the occurrence and management of adverse events. Less experienced teams may fail to recognize or appropriately manage minor events, allowing them to escalate into more serious complications. This variability in expertise represents a potential confounding factor. Second, most cases of device dysfunction were related to human error, further highlighting the need for training on the use of oxygenation devices (whether standard oxygen, HFNC, non-invasive ventilation, intubation), supported by clear protocols and systematic checklists [26–28]. Third, unlike other studies, we focused exclusively on respiratory complications as endpoints. While hemodynamic parameters were not primary endpoints, they were monitored, and no hemodynamic complications were observed. Fourth, we followed patients for only 5 min after the end of transport. Although most observed severe and non-severe adverse events may have appeared transient or benign, their potential impact on longer-term, patient-centered outcomes requires further research. While HFNC is less invasive than intubation, patients with acute respiratory distress requiring HFNC are clinically unstable. Any complication during or after transport can have serious consequences. However, we anticipate that distinguishing between complications arising from transport and those due to disease progression will be challenging. Fifth, we did not assess neurological status during the transport, preventing us from determining whether the most agitated patients were more prone to cannula pullouts. Sixth, there was no comparison with other oxygenation modalities. Finally, the financial and environmental costs of using HFNC were not evaluated, but this aspect warrants future consideration.

## Conclusion

This study demonstrates that intra-hospital transport of adult critically ill patients on HFNC can be performed with a relatively low incidence of severe adverse events. Non-severe adverse events were more frequent (23% of cases), but their clinical impact after transport could not be assessed in this study and warrants further investigation. These findings highlight that transport under HFNC is feasible but should be approached with caution, requiring a well-trained team, clear protocols, and optimized monitoring to ensure safety.

## Supplementary Information

**Additional file 1.**

## Data Availability

The study data will be shared with investigators upon reasonable request to the corresponding author.

## Abbreviations

BMI: Body mass index
CT: Computed tomography
ED: Emergency department
ICU: Intensive care unit
IQR: Interquartile range
FiO_2_: Fraction oxygen inspired
HFNC: High flow nasal cannula
MRI: Magnetic resonance imaging
PaCO_2_: Arterial pressure of dioxide of carbon
PaO_2_: Arterial pressure of oxygen
SAPS II: Simplified acute physiology score
SpO_2_: Pulse oxygen saturation
