# Supplementary material for: Intra-hospital transport of adult critically ill patients treated with high flow nasal cannula oxygen: a prospective observational multicenter study
Source: Ann Intensive Care. 2025 Jul 8;15:93. doi: 10.1186/s13613-025-01502-7 (PMC12238688; doi:10.1186/s13613-025-01502-7)
Supplement: Supplementary file 1 — Additional file 1. [file 13613_2025_1502_MOESM1_ESM.docx]

**Appendix**

**eFigure1 A. Photograph of transportable HFNC with energy pack with a 6-hour battery life**

**
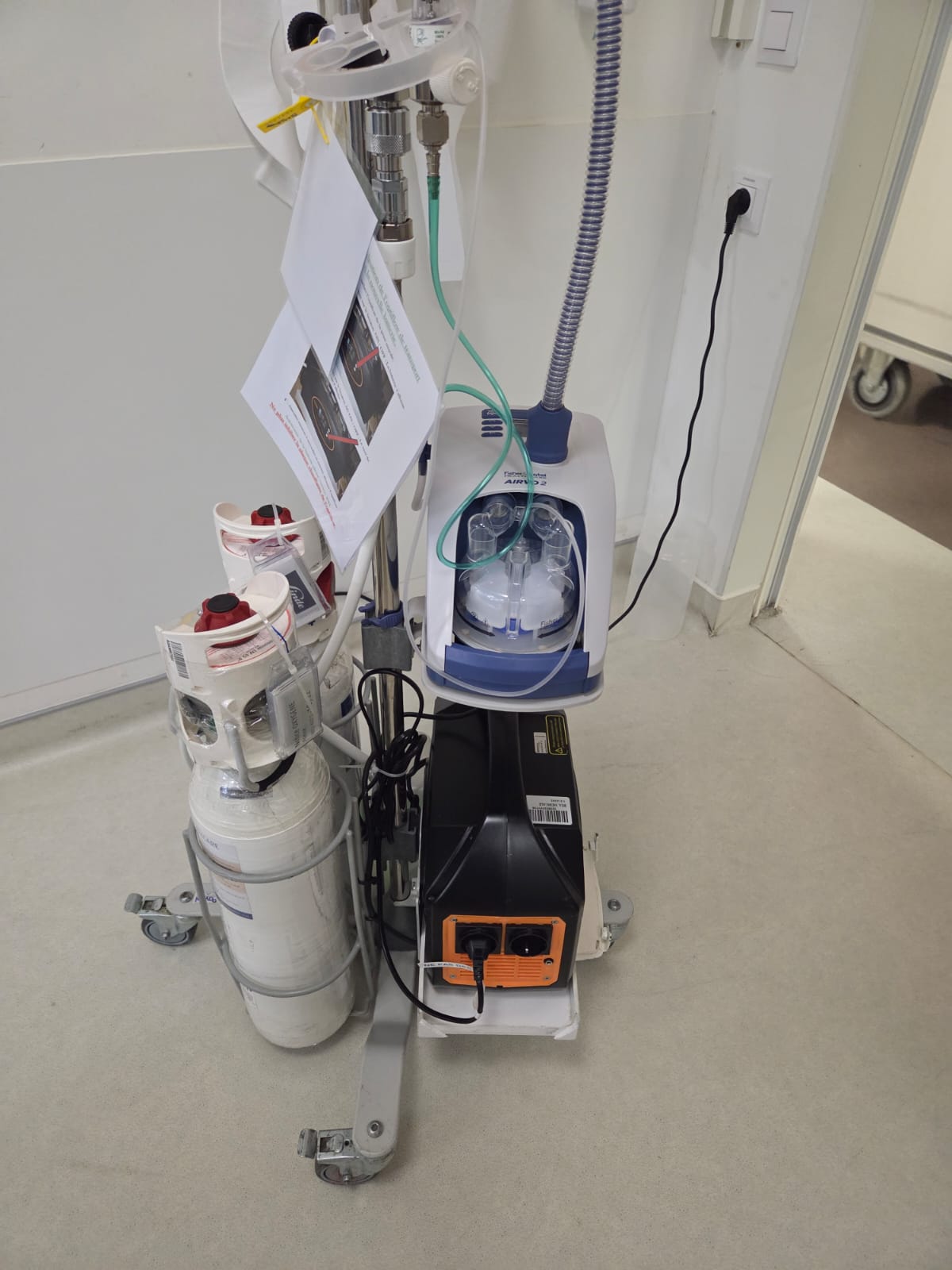
**

**eFigure1 B. Photograph of transportable HFNC with specific battery**

**
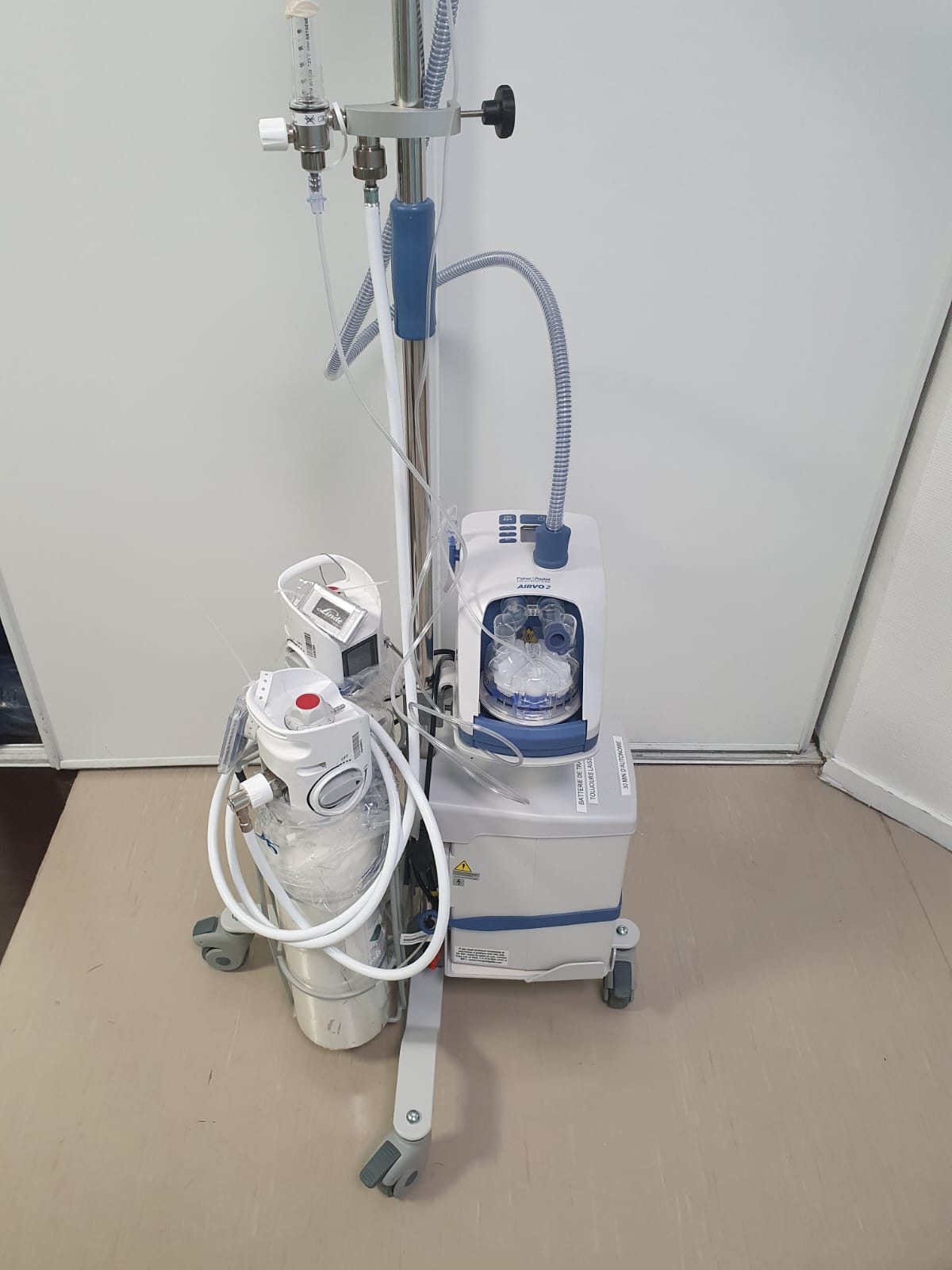
**

**eTable1: Severity of patients during all the transports and indications for all the transport**

|  | N=187 |
| --- | --- |
| **Baseline SpO_2_/FiO_2_ ratio before transport, n (%)** | |
| SpO_2_/FiO_2_ ≤  235, n (%) SpO_2_/FiO_2_ > 235 and ≤  315, n (%) SpO_2_/FiO_2_ > 315, n (%) | 143 (76.5%) 35 (18.7%)  9 (4.8) |
| **Initial SpO_2_/FiO_2_ before transport, median (IQR)** | |
| ICU–Radiology round‑trip for imaging  From ED or ward to ICU  Other transfers* | 196 (157 – 241)  139 (100 – 167)  216 (187 – 239) |
| **Initial ROX index before transport, median (IQR)** | |
| ICU–Radiology round‑trip for imaging  From ED or ward to ICU,  Other transfers* | 8.4 (6.2 – 10.7) 5.2 (4.1 – 7.6) 9.5 (8.4 – 11.3) |
| **Indication of transport, n (%)** |  |
| ICU–Radiology round‑trip for imaging  From ED or ward to ICU  Other transfers*^,£^ | 92 (49.2)  56 (29.9)  39 (20.9) |

SpO_2_: pulse oxygen saturation of oxygen; FiO_2_, inspired fraction of oxygen; ICU, intensive care unit; IQR, interquartile range; ED, emergency department

* Included coronary angiography, radiography, to or from operating room, doppler ultrasound, ICU to medical ward, and only one magnetic resonance imaging (MRI).

^£^ In the case of transport for MRI: HFNC was used only during the transfer to the MRI preparation room, after which the HFNC was switched to standard oxygen (HFNC is not compatible with MRI) and the transport HFNC was reused once the examination had been completed.

**eTable2: Characteristics of the following transports**

| **Indication of 2^nd^ transport** | **Patients (N= 18)** |
| --- | --- |
| CT scan, n (%) | 6 (33.3) |
| ICU to medical ward, n (%) | 9 (50.0) |
| Others, n (%)* | 3 (16.6) |
| **Indication of 3^rd^ transport** | **Patients (N= 3)** |
| ICU to operating room, n (%) | 1 (33.3) |
| ICU to coronary angiography, n (%) | 1(33.3) |
| Coronary angiography to ICU, n (%) | 1 (33.3) |
| **Indication of 4th transport** | **Patient (N=1)** |
| CT scan, n (%) | 1 (100) |
| **Most recent arterial blood gas before the 2^nd^ transport, (%)** | **Patients (N=18)** |
| pH, median (IQR) | 7.47 (7.42 - 7.50) |
| PaO_2_ (mmHg), median (IQR) | 77.50 (63.5 - 91.3) |
| PaCO_2_ (mmHg), median (IQR) | 35.5 (31.5 - 39.3) |
| FiO_2_ (%), median (IQR) | 50 (40 - 60) |
| Flow rate of HFNC (L/min), median (IQR) | 50 (40 - 60.00) |

CT, computed tomography; ICU, intensive care unit; PaO_2_, arterial partial pressure of oxygen; PaCO_2_, arterial partial pressure of carbon dioxide; FiO_2_, inspired oxygen fraction; HFNC, nasal high-flow canula

* One patient had a coronarography, one had a doppler ultrasound examination, and one had a magnetic resonance imaging (MRI); in this case, the patient was transferred under transportable HFNC, but standard oxygen was used during the MRI.

**eTable 3. Number of any (serious or non-serious) adverse events per patient**

| **Number of adverse events** | **Number of patients** |
| --- | --- |
| *Across all transports* | |
| 0 | 131 |
| 1 | 27 |
| 2 | 6 |
| 3 | 1 |
| 4 | 1 |
| 5 | 1 |
| *During the first transport* | |
| 0 | 131 |
| 1 | 27 |
| 2 | 4 |
| 3 | 2 |
| 4 | 1 |

**eTable4. FiO₂ increase during imaging transports vs. other transports for all transports**

|  | **Transport with or without at least one**  **increase of FiO_2_** | |
| --- | --- | --- |
|  | **No increase of FiO_2_** | **Increase of FiO_2_** |
| From ED or ward to ICU | **54** | **2** |
| ICU–Radiology round‑trip for imaging | **68** | **24** |
| Other transfers | **36** | **3** |

Global Fisher test: p-value = 0.0002

Transport for imaging vs. the other 2 categories: p-value <0.0001

**eTable5. Number of patients with at least one desaturation episode (SpO₂ <90%) during transport:**

| ***Across all transports (187 transports****):* | |
| --- | --- |
| From ED or ward to ICU | 7/56 |
| ICU–Radiology round‑trip for imaging (CT/PET) | 15/84 |
| Other transfers | 5/25 |
| Total | 27/165 |
| ***During the first transport (165 transports):*** | |
| From ED or ward to ICU | 7/56 |
| ICU–Radiology round‑trip for imaging (CT/PET) | 15/84 |
| Other transfers | 3/25 |
| Total | 25/165 |

CT: computed tomography; ED: emergency department; ICU: intensive care unit; PET: positron emission tomography

**eFigure 2. Evolution of systolic and diastolic arterial pressure during transport
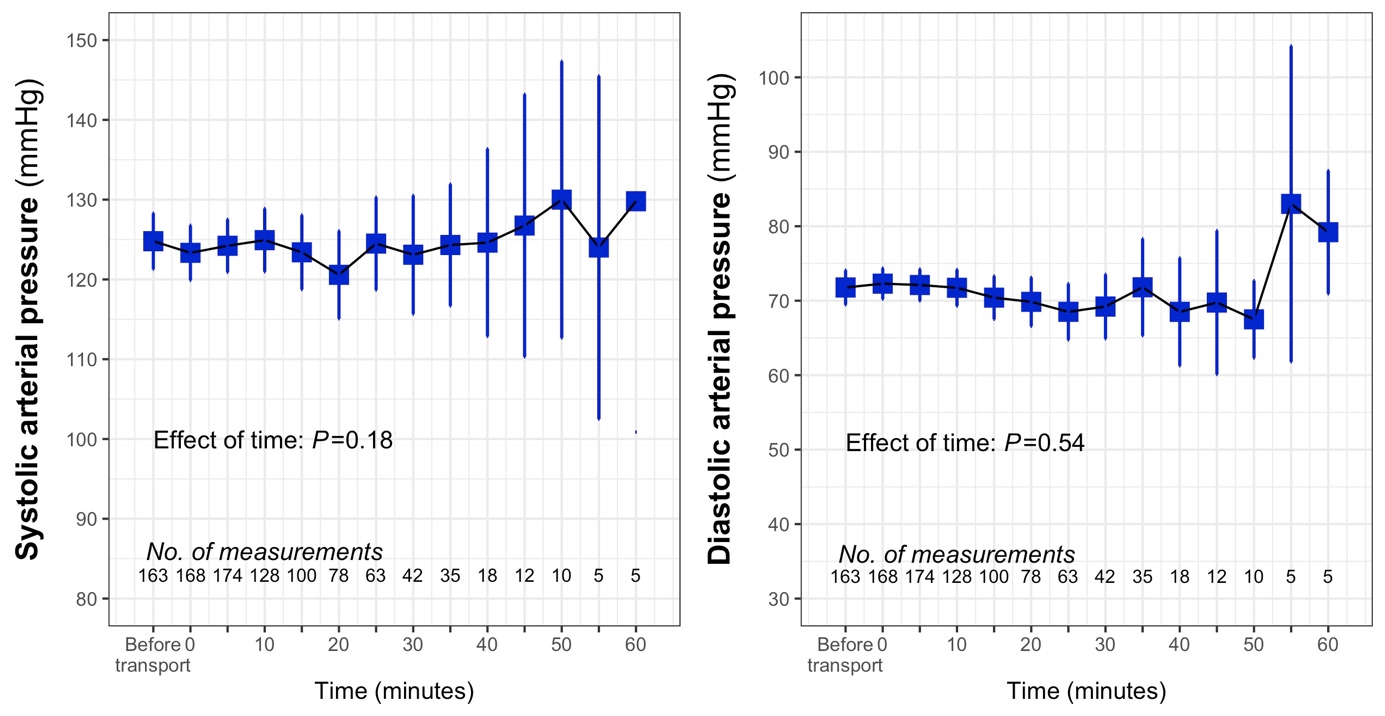
**

**eTable6. Search for factors associated with the occurrence of any adverse event during first transport — Bivariate analysis***

|  | **No adverse event** | **Occurrence of at least one adverse event** | **P value** |
| --- | --- | --- | --- |
| **Sex** |  |  | 0.70 |
| Female | 88 | 21 |  |
| Male | 43 | 13 |  |
| **Age (years)** | 69 [57-74] | 70 [61-76] | 0.41 |
| **SAPS II** | 40 [32-51] | 43 [35-52] | 0.49 |
| **SOFA score prior to transport** | 3 [2-4] | 3 [2-5] | 0.96 |
| **Body mass index (kg/m²)** | 26 [23-30] | 27 [24-30] | 0.50 |
| **Centre** |  |  | P values for two-by-two between-center difference all> 0.20 |
| Dax | 21 | 5 |  |
| Martigues | 2 | 0 |  |
| Orléans | 96 | 25 |  |
| Vannes | 12 | 4 |  |
| **Indication for transport** |  |  | P=0.001 for Imaging vs from the ED |
| From ED or ward to ICU | 52 | 4 |  |
| ICU–Radiology round‑trip for imaging (CT/PET) | 56 | 28 |  |
| Other transfers | 23 | 2 |  |
| **Initial SpO_2_/FiO_2_** | 187 [138-224] | 196 [132-240] | 0.67 |
| **Categories of initial SpO_2_/FiO_2_** |  |  | All p values >0.20 |
| ≤235 | 103 | 24 |  |
| >235≤315 | 22 | 8 |  |
| >315 | 6 | 2 |  |
| **Initial ROX index** | 7.8 [5.1-9.7] | 7.0 [5.4-10.1] | 0.99 |
| **Initial PaCO_2_ (mmHg) (2 missing)** | 37 [32-43] | 36 [32-50] | 0.15 |
| **Initial PaCO_2_> 45 mmHg (2 missing)** |  |  | 0.85 |
| No | 102 | 27 |  |
| Yes | 29 | 7 |  |
| **Surgery within 24hr** |  |  | 0.006 |
| No | 130 | 31 |  |
| Yes | 1 | 3 |  |
| **Post-operative admission** |  |  | 0.14 |
| No | 127 | 31 |  |
| Yes | 4 | 3 |  |
| **Acute resp failure** |  |  | 0.85 |
| No | 25 | 6 |  |
| Yes | 106 | 28 |  |
| **Acute on chronic resp failure** |  |  | 0.60 |
| No | 102 | 25 |  |
| Yes | 29 | 9 |  |
| **Septic shock** |  |  | 0.34 |
| No | 125 | 31 |  |
| Yes | 6 | 3 |  |
| **Acute renal failure** |  |  | 0.01 |
| No | 124 | 28 |  |
| Yes | 7 | 6 |  |
| **Lung infection** |  |  | 0.70 |
| No | 47 | 11 |  |
| Yes | 84 | 23 |  |
| **Smoking** |  |  | 0.94 |
| No | 101 | 26 |  |
| Yes | 30 | 8 |  |
| **COPD** |  |  | 0.86 |
| No | 102 | 26 |  |
| Yes | 29 | 8 |  |
| **Asthma** |  |  | 0.75 |
| No | 117 | 31 |  |
| Yes | 14 | 3 |  |
| **Sleep apnea** |  |  | 0.35 |
| No | 119 | 29 |  |
| Yes | 12 | 5 |  |
| **Home non-invasive ventilation** |  |  | 0.95 |
| No | 119 | 31 |  |
| Yes | 12 | 3 |  |
| **Home oxygen therapy** |  |  | 0.28 |
| No | 120 | 33 |  |
| Yes | 11 | 1 |  |
| **Hypertension** |  |  | 0.63 |
| No | 67 | 19 |  |
| Yes | 64 | 15 |  |
| **Ischemic cardiomyopathy** |  |  | 0.62 |
| No | 107 | 29 |  |
| Yes | 24 | 5 |  |
| **Arial fibrillation** |  |  | 0.18 |
| No | 112 | 32 |  |
| Yes | 19 | 2 |  |
| **Chronic kidney disease** |  |  | 0.61 |
| No | 116 | 29 |  |
| Yes | 15 | 5 |  |
| **Cirrhosis** |  |  | 0.44 |
| No | 127 | 32 |  |
| Yes | 4 | 2 |  |
| **Immunosuppression** |  |  | 0.11 |
| No | 99 | 30 |  |
| Yes | 32 | 4 |  |

COPD : chronic obstructive pulmonary disease ; ED, emergency department ; FiO_2_, inspired fraction of oxygen; ICU, intensive care unit ; SOFA, sequential organ failure assessment; SAPS II, simplified acute physiology score; SpO_2_, pulse oxygen saturation of oxygen

* : The association between each patient’s characteristic and the occurrence of any adverse event (either serious or not) was searched by bivariate logistic regression analysis. Each characteristic presents more than 5% of the study population or accounting for more than 5% of all adverse events, and associated with the dependent variable at P < 0.2 in were included in a multivariable logistic regression model in a single step (see the Multivariable analysis 1 in the next table).

**eTable7** : **Factors associated with the occurrence of any adverse event during first transport — Multivariable analysis 1**

|  | **Odds-ratio (95%CI)** | **P-value** |
| --- | --- | --- |
| PaCO_2_ before transport (for 1-mmHg increase) | 1.01 (0.98 to 1.05) | 0.46 |
| Acute renal failure | 3.3 (0.83 to 13.05) | 0.09 |
| Immunosuppression | 0.37 (0.11 to 1.24) | 0.108 |
| Atrial fibrillation | 0.3 (0.06 to 1.44) | 0.13 |
| Surgery within 24 hours after ICU admission | 5.98 (0.53 to 67.97) | 0.15 |
| Transport for imaging (vs transport from the ED or the ward) | 5.66 (1.79 to 17.88) | **0.003** |
| Other transports (vs transport from the ED or the ward) | 1.13 (0.19 to 6.81) | 0.89 |

**eTable8** : **Factors associated with the occurrence of any adverse event during first transport — Multivariable analysis 2**

|  | **OR (95%CI)** | **P-value** |
| --- | --- | --- |
| Age (for 1-yr increase) | 1 (0.97 to 1.04) | 0.83 |
| SAPS II (for 1-point increase) | 1.02 (0.99 to 1.06) | 0.23 |
| PaCO_2_ before transport (for 1-mmHg increase) | 1.03 (0.99 to 1.07) | 0.17 |
| Initial SpO_2_/FiO_2_ ratio (for 1-point increase) | 1 (0.99 to 1.01) | 0.64 |
| ROX index | 0.99 (0.81 to 1.21) | 0.93 |
| Acute renal failure | 3.23 (0.77 to 13.56) | 0.11 |
| Immunosuppression | 0.34 (0.09 to 1.21) | 0.10 |
| Atrial fibrillation | 0.41 (0.08 to 2.07) | 0.28 |
| Surgery within 24 hours after ICU admission | 5.93 (0.52 to 67.44) | 0.15 |
| Transport for imaging (vs transport from the ED) | 5.91 (1.76 to 19.89) | **0.004** |
| Other transports (vs transport from the ED) | 1.19 (0.17 to 8.45) | 0.864 |

This multivariable analysis used the same independent variables than the first multivariable analysis and in addition was adjusted for variables supposed to reflect the patient’s severity of disease and the severity of respiratory impairment (Age, SAPS II, SpO_2_/FiO_2_ ratio, and ROX index).
